# Acceptability of Home-Based HIV Care Offered by Community Health Workers in Tshwane District, South Africa: A Survey

**DOI:** 10.1089/apc.2021.0216

**Published:** 2022-02-10

**Authors:** Sanele Ngcobo, Theresa Rossouw

**Affiliations:** ^1^ Department of Family Medicine and University of Pretoria, Pretoria, South Africa.; ^2^ Department of Immunology, University of Pretoria, Pretoria, South Africa.

**Keywords:** community health workers, home-based HIV care, acceptability, disclosure

## Abstract

Human immunodeficiency virus (HIV) remains the biggest public health challenge faced by South Africa (SA). To alleviate overcrowding in health facilities, ward-based primary health care outreach teams, consisting of community health workers (CHWs) led by a nurse, were introduced. The aim of this study was to assess the acceptability of community-based HIV services offered by CHWs. A survey was conducted in 10 clinics across Tshwane district, Gauteng, SA, between November 2020 and May 10, 2021. CHWs conducted interviewer-administered standardized questionnaires with 674 adult participants. Overall, 95.5% of participants thought that home-based HIV care is a good initiative and rated screening for illnesses and referral to health facilities highly. Although the vast majority (>94%) were willing to disclose their status to health professionals in clinics, women were more willing to do so. Only 53.6% of participants were willing to disclose their HIV status to a CHW from the same neighborhood and 28.8% would find it problematic if CHWs visited them at home with branded cars. Participants had different preferences, mostly determined by region, how long they had been on antiretroviral treatment, whether they had been informed about CHWs, age, and gender. More work is needed to understand and accommodate regional differences and individual preferences.

## Introduction

South Africa (SA) currently faces a quadruple burden of diseases: maternal, new-born, and child-related illness; human immunodeficiency virus (HIV) and tuberculosis (TB); noncommunicable diseases; and violence and injury. Of these, HIV is arguably still the biggest public health challenge facing the country. In 2020, Statistics SA estimated that the HIV prevalence was ∼13% in the general population, 19.1% [95% confidence interval (CI): 12.1–24.7] in adults aged 15–49 years, with the total number of people living with HIV (PLWHIV) estimated to be ±7.8 million.

In addition, 79,625 AIDS-related deaths were recorded in 2020.^1^ Enrolling and keeping PLWHIV on antiretroviral treatment (ART) is a key factor in reducing HIV-related mortality and HIV transmission.^2^ In SA, only 72% (95% CI: 48–93) of PLWHIV were on ART in 2020,^3^ and this proportion was even lower in Gauteng province and Tshwane district: 56% and 55.4%, respectively.^4^ To effectively respond to this quadruple burden of diseases in general, and HIV in particular, SA had to move away from a passive, vertical, disease-specific, and individually oriented health system to an integrated, preventative, and population-oriented health system.^5^

Accordingly, the SA government started a process of re-engineering the primary health care sector with the ultimate aim of increasing access to quality health care for all.^5^ Ward-based primary health care outreach teams (WBPHCOTs) are one of the key components of this re-engineered sector. These teams consist of community health workers (CHWs), led by a nurse who is linked to a primary health care facility.^6^ In a qualitative study from United States, participants receiving HIV care from CHWs reported that CHWs were more caring and supportive, met participants where they were located, and had more time for interaction.^7^ In another study from Mexico, CHWs were influential in educating participants about HIV and ART, linkage to care, and ART adherence.^8^

The SA Department of Health recently introduced a scope of work for CHWs. In terms of HIV-related care, CHWs are tasked with various prevention activities, such as distributing condoms, promoting voluntary medical male circumcision, and providing information about pre-exposure prophylaxis. In the treatment realm, CHWs are expected to promote the disclosure of HIV status, trace patients who have become lost to follow-up, and provide adherence support. For these activities to be successful, there is a need to understand if they will be accepted by PLWHIV and, if so, what preferences they have for receiving these services at a household level. This study is an extension of a qualitative study that looked at perceived barriers and benefits (by WBPHCOTs and PLWHIV) of implementing HIV care at the community level in Tshwane district.^9^ The aim of this study was to assess the acceptability of community-based HIV services offered by CHWs to PLWHIV.

## Methods

### Overview

**FIG. 1.**
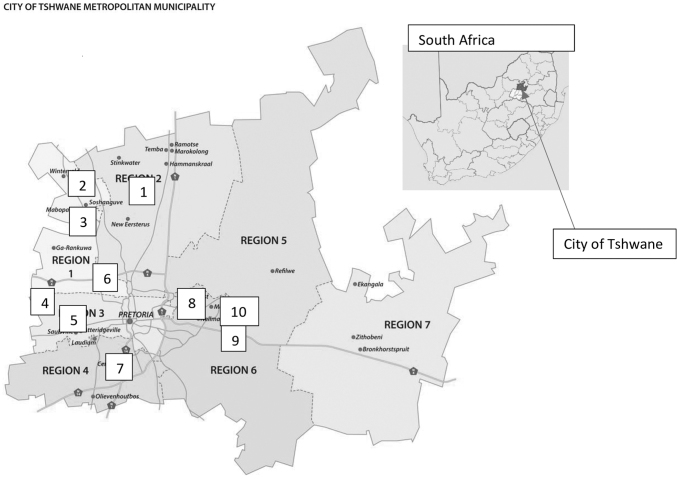
Tshwane regions (Clinics are numbered from 1 to 10).

We conducted a survey in 10 clinics in the 7 Tshwane regions (Fig. 1) in Gauteng, SA, between November 2020 and May 2021. Region seven was excluded from the analysis due to nonresponse. Since patient satisfaction can be influenced by patient volume,^10^ clinics were purposefully selected based on the number of PLWHIV seen on a monthly basis. A combination of high (≥3,500 PLWHIV on ART, *n* = 5) and low volume (<3,500 PLWHIV on ART, *n* = 5) clinics were selected. Trained CHWs completed 674 structured interviewer-administered questionnaires, with participants answering questions related to home-based HIV care.

Questionnaires were in English, but the CHWs, who are conversant with the local languages in the area, ensured that all participants clearly understood the questions asked. Questionnaires were completed with smartphones using the Qualtrics online survey platform. The principal investigator and research assistant conducted a 4-h practical training session with CHWs. This training included an overview of the Qualtrics online survey platform; research ethics, such as the importance of confidentiality and informed consent; and the questionnaire itself.

The questionnaire was designed based on the themes that had emerged from the focus group discussions during the prior study.^9^ The questionnaire covered sociodemographic information, the role of CHWs, CHW services, HIV status disclosure, and CHW home visits. The questionnaire was piloted in one of the sites among 20 PLWHIV and minor changes were made to improve the comprehension of some questions. Completed questions were imported to the Qualtrics online survey platform (University of Pretoria).

### Inclusion and exclusion criteria

Only adult (≥18 years) PLWHIV currently on ART at one of the 10 clinics were included in this study. Participants who were unable to provide informed consent (i.e., too ill) were excluded from the study.

### Participant recruitment

From November 1, 2020 to May 10, 2021, 64 trained CHWs recruited participants among PLWHIV attending one of the 10 clinics for routine HIV care. All potential participants were invited to participate in the survey and were selected according to the inclusion and exclusion criteria. Informed consent was obtained electronically from each individual participant.

### Interview procedure

CHWs informed PLWHIV about the study, explained the entire process, and assured them about the voluntary nature of participation and confidentiality. All participants were provided with a standard definition of CHWs to ensure a shared understanding. This definition was: “A community health worker is a member of a community who is linked to a local clinic and provides basic health and medical care services within their community under the supervision of a nurse. They work in the community to help people live a healthy lifestyle, check and assist those with illnesses to take their medication, educate them about their illnesses and link them to clinics if needed. They work together in ward-based outreach teams.”^11^

Once participants understood what a CHW was, they continued with the interview.

### Data analysis

Data were cleaned and exported from the Qualtrics online survey platform (University of Pretoria) to SPSS version 26 (IBM Corp., 2019) for analysis.^12^ Using “identify duplicate cases wizard” on SPSS, we identified two duplicates using participants' file numbers and these duplicates were excluded from the analysis. Data analysis was conducted quantitatively: frequencies, proportions, means, and standard deviations (SDs) were calculated and compared according to region, patient volume, age, gender, duration on treatment, and information provided about CHWs and their role.

Studies from Zambia, Kenya, and Tanzania found that levels of internalized stigma (whereby affected individuals endorse stereotypes, anticipate social rejection, and believe they are devalued members of the society)^13^ decreased 2 years after treatment initiation^14–16^ and we, therefore, categorized treatment duration into <2 or ≥2 years in our analysis. We also assessed the association between disclosure to CHWs in the community and gender, duration on treatment, and age using logistic regression and the Kruskal–Wallis H test.

### Ethical considerations

The Research Ethics Committee of the Faculty of Health Sciences of the University of Pretoria granted ethics approval for the study (reference number 580/2018). Permission to collect data was obtained from Tshwane health district and all facilities involved. Each participant gave informed consent before participating in the study. No personal identifying information was captured.

## Results

Results of this survey are presented according to the following four areas: description of participants, HIV status disclosure, home visits, and home-based HIV services. There were no differences observed between high volume and low volume clinics, so this analysis is not included in the results.

### Description of participants

A total of 674 participants, with a mean age of 38.2 years (SD ±11.1), were interviewed in 10 clinics (Table 1). These clinics were spread across six regions: two in region 1, one in region 2, four in regions 3 and 4, and three in regions 5 and 6. A total of 500 participants (76.5%) were female, whereas male participants made up less than a quarter (*n* = 154; 23.5%) of the participants. Although more than half of the participants in all the regions were women, this was especially notable in region 1 where women accounted for 95.2% (*n* = 119) of the participants. The majority of participants (*n* = 406; 63.1%) had been on ART for ≥2 years. Notably, regions 5 and 6 were the only regions dominated by participants who had been on treatment for <2 years.

**Table 1.** Description of Participants Involved per Region in Tshwane

	Total	Region 1 (*N* = 126)* n *(%)	Region 2 (*N* = 228)* n *(%)	Regions 3 and 4*^a^* (*N* = 337)* n *(%)	Regions 5 and 6*^a^* (*N* = 83)* n *(%)
Age mean (SD)	38.2 (11.1)	32.6 (6.9)	40.4 (11.3)	39.2 (10.8)	38.2 (13.6)
Gender^b^					
Female	500 (76.5%)	120 (95.2%)	162 (72.0%)	163 (74.1%)	55 (66.3%)
Male	154 (23.5%)	6 (4.8%)	62 (28.0%)	57 (25.9%)	28 (33.7%)
Duration of ART (years)					
<2	237 (36.9%)	38 (30.4%)	60 (26.8%)	77 (36.3%)	62 (75.5%)
≥2	406 (63.1%)	87 (69.6%)	164 (73.2%)	135 (63.7%)	20 (24.4%)

^a^ These regions are very close to one another, they are usually combined when reporting and they had very low numbers when separated.

^b^ Twenty participants did not have gender indicated.

ART, antiretroviral treatment; *n* = number; SD, standard deviation.

### HIV status disclosure

**FIG. 2.**
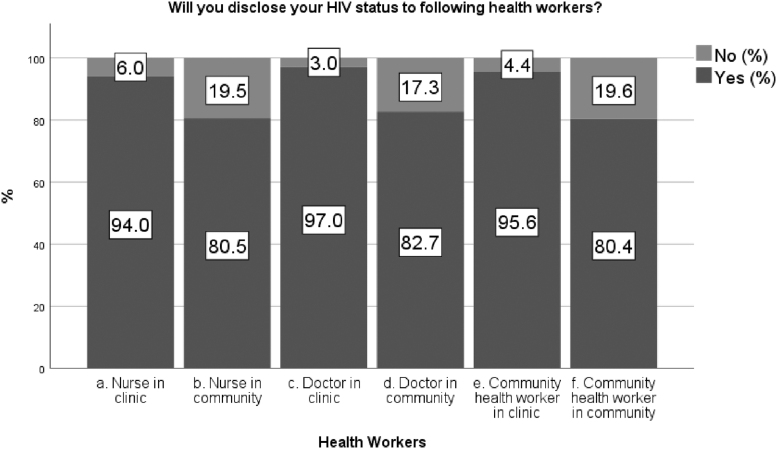
Disclosure of HIV status to health workers. HIV, human immunodeficiency virus.

Asked if they were willing to disclose their status to health professionals working in clinics, 94.0%, 95.6%, and 97.0% participants indicated that they were willing to disclose their status to nurses, CHWs, and doctors, respectively (Fig. 2). Fewer participants were willing to disclose their status to nurses, CHWs, and doctors in the community: 80.5%, 80.4%, and 82.7%, respectively (Table 2). There was no difference in the mean age of participants willing to disclose their status to nurses, doctors, and CHWs in the community: 38.3 years (SD ±10.9), 38.2 years (SD ±11.0), and 38.3 years (SD ±11.0), respectively.

**Table 2.** HIV Status Disclosure to Nurses, Doctors, and Community Health Workers at a Facility and Community Level

	Total* n *(%)	Duration on treatment* n *(%)		Gender* n *(%)		Informed about CHWs and their role* n *(%)		
		<2 years (A)	≥2 years (B)	Female (A)	Male (B)	No (A)	Yes (B)	Not sure (C)
Nurse in clinic								
No	37 (6.0)	12 (5.5)	20 (5.3)	19 (4.1)	14 (9.9)A	8 (3.7)	26 (6.9)	3 (11.5)
Yes	584 (94.0)	206 (94.5)	357 (94.7)	442 (95.9)B	127 (90.1)	207 (96.3)	352 (93.1)	23 (88.5)
Nurse in community								
No	109 (19.5)	56 (28.4)B	47 (13.7)	80 (18.9)	26 (21.0)	54 (27.6)B	50 (14.6)	4 (20.0)
Yes	451 (80.5)	141 (71.6)	297 (86.3)A	344 (81.1)	98 (79.0)	142 (72.4)	292 (85.4)A	16 (80.0)
Doctor in clinic								
No	16 (3.0)	6 (3.1)	10 (3.0)	11 (2.6)	5 (4.4)	6 (3.1)	10 (3.1)	01 (0.0)
Yes	523 (97.0)	186 (96.9)	321 (97.0)	407 (97.4)	109 (95.6)	188 (96.9)	314 (96.9)	191 (100.0)
Doctor in community								
No	89 (17.3)	49 (27.4)B	38 (11.9)	68 (17.3)	21 (18.4)	50 (26.2)B	34 (11.3)	5 (26.3)
Yes	424 (82.7)	130 (72.6)	281 (88.1)A	324 (82.7)	93 (81.6)	141 (73.8)	267 (88.7)A	14 (73.7)
CHW in clinic								
No	22 (4.4)	7 (3.9)	15 (4.9)	13 (3.4)	9 (8.0)A	8 (4.4)	12 (4.0)	1 (5.3)
Yes	480 (95.6)	172 (96.1)	293 (95.1)	372 (96.6) B	103 (92.0)	175 (95.6)	286 (96.0)	18 (94.7)
CHW in community								
No	102 (19.6)	54 (28.6)B	46 (14.5)	75 (18.8)	27 (22.9)	56 (29.0)B	40 (13.2)	5 (21.7)
Yes	419 (80.4)	135 (71.4)	272 (85.5)A	323 (81.2)	91 (77.1)	137 (71.0)	263 (86.8)A	18 (78.3)
CHW from your neighborhood								
No	257 (46.4)	99 (54.1)B	146 (42.4)	188 (45.1)	57 (48.3)	115 (59.0)B	132 (38.9)	10 (52.6)
Yes	297 (53.6)	84 (45.9)	198 (57.6)A	229 (54.9)	61 (51.7)	80 (41.0)	207 (61.1)A	9 (47.4)
CHW not from neighborhood								
No	110 (18.5)	33 (16.6)	69 (18.7)	77 (17.1)	28 (22.0)	41 (19.5)	67 (18.4)	2 (10.0)
Yes	485 (81.5)	166 (83.4)	300 (81.3)	372 (82.9)	99 (78.0)	169 (80.5)	297 (81.6)	18 (90.0)

Results are based on two-sided tests. For each significant pair, the key of the category with the smaller column proportion appears in the category with the larger column proportion.

Significance level (A, B, C): <0.05′′

Tests are adjusted for all pairwise comparisons within a row of each innermost sub-table using the Bonferroni correction.

CHW, community health worker; HIV, human immunodeficiency virus; *n*, number.

Women were significantly more willing to disclose their HIV status to nurses and CHWs at the clinic than men: 95.9% versus 90.1% (*p* < 0.01) and 96.6% versus 92.0% (*p* = 0.035), respectively (Table 2). Participants who had been on treatment for <2 years were significantly less likely to disclose their HIV status to nurses (71.6% vs. 86.3%; *p* < 0.01), doctors (72.6% vs. 88.1%; *p* < 0.01), and CHWs (71.4% vs. 85.5%; *p* < 0.01) at a community level, but not at clinic level, when compared with those who had been on treatment for ≥2 years. Participants who had been informed about CHWs and their role when they started treatment were significantly more likely to disclose their status to nurses (85.4% vs. 72.4%; *p* < 0.01), doctors (88.7% vs. 73.8%; *p* < 0.01), and CHWs (86.8% vs. 71.1%; *p* < 0.01) at a community level.

Willingness to disclose differed significantly among regions. For instance, participants from region 1 (compared with regions 4 and 5) were significantly more likely to disclose their HIV status to nurses (90.2% vs. 55.7%; *p* < 0.01), doctors (91.0% vs. 53.7%; *p* < 0.01), and CHWs (87.6% vs. 53.4%; *p* < 0.01) at a community level. Similar patterns were observed between region 2 (compared with regions 4 and 5; Supplementary Tables S1 and S2). In multi-variable logistic regression, only the association between disclosure to CHWs at the community level and region remained statistically significant with an odds ratio (OR) of 0.4 (95% CI: 0.3–0.6, *p* < 0.01; Supplementary Table S3).

Participants were much less willing to disclose their HIV status to a CHW who was from the same neighborhood, with only 53.6% of participants overall agreeing to this option, compared with 81.5% who would disclose to someone from a different neighborhood (Table 2 and Supplementary Table S4). Participants who had been on treatment for <2 years were significantly less likely to be willing to disclose to a CHW from the same neighborhood (45.9% vs. 57.6% on treatment for ≥2 years; *p* < 0.01) as were participants who had not been informed about CHWs when diagnosed (41.0% vs. 61.1% of those who had been informed; *p* < 0.01). The main reasons (from prespecified responses) for unwillingness to disclose to CHWs who were from the same neighborhood were: “I don't trust my neighbours” (42.4%; *n* = 118); “I don't like to talk about my personal things with my neighbours” (31.3%; *n* = 87); and “I am afraid that they will tell other people about my HIV status” (16.9%; *n* = 47; Supplementary Table S5)

### Home visits

The majority of participants (*n* = 580; 89.4%) would like to be visited by a CHW; however, those who had been on treatment for ≥2 years were significantly more comfortable with this option: 91.4% versus 85.8% (*p* < 0.01; Table 3 and Supplementary Table S6). Participants who had been informed about CHWs were also more comfortable with home visits than their uninformed counterparts: 94.4% versus 81.7% (*p* < 0.01). Participants from region 1 were more comfortable with home visits than participants from regions 5 and 6: 94.4% versus 73.5% (*p* < 0.01). There was no significant difference regarding willingness to be visited by CHWs between males and females or between different age groups.

**Table 3.** Home Visit by a Community Health Worker

	Total* n *(%)	Duration on treatment* n *(%)		Gender* n *(%)		Informed about CHWs and their role* n *(%)		
		<2 years (A)	≥2 years (B)	Female (A)	Male (B)	No (A)	Yes (B)	Not sure (C)
Would you like to be visited by a CHW where you stay?								
No	69 (10.6)	32 (14.2)B	34 (8.6)	46 (9.5)	20 (13.6)	42 (18.3)B	22 (5.6)	4 (16.0)
Yes	580 (89.4)	193 (85.8)	361 (91.4)A	436 (90.5)	127 (86.4)	187 (81.7)	371 (94.4)A	21 (84.0)
How often should a CHW visit you at home?								
Weekly	144 (21.6)	43 (18.2)	86 (21.4)	101 (20.4)	28 (18.5)	30 (12.7)	110 (27.4)A	4 (15.4)
Monthly	383 (57.5)	132 (55.9)	241 (60.0)	300 (60.6)	82 (54.3)	137 (58.1)	228 (56.7)	17 (65.4)
Every 6 months	65 (9.8)	26 (11.0)	38 (9.5)	46 (9.3)	18 (11.9)	23 (9.7)	40 (10.0)	2 (7.7)
Yearly	11 (1.7)	2 (0.8)	9 (2.2)	8 (1.6)	3 (2.0)	3 (1.3)	7 (1.7)	01 (0.0)
Never	63 (9.5)	33 (14.0)B	28 (7.0)	40 (8.1)	20 (13.2)	43 (18.2)B	17 (4.2)	3 (11.5)
Should CHWs wear a uniform?								
No	101 (16.3)	34 (15.6)	64 (17.2)	74 (15.9)	24 (17.5)	59 (27.6)B	37 (9.6)	5 (23.8)
Yes	519 (83.7)	184 (84.4)	309 (82.8)	390 (84.1)	113 (82.5)	155 (72.4)	347 (90.4)A	16 (76.2)
Should CHWs come to your house with branded cars?								
No	410 (71.2)	134 (69.1)	253 (71.9)	310 (71.9)	88 (69.8)	120 (62.5)	279 (75.8)A	10 (66.7)
Yes	166 (28.8)	60 (30.9)	99 (28.1)	121 (28.1)	38 (30.2)	72 (37.5)B	89 (24.2)	5 (33.3)
Should CHW to come to your house if you missed your clinic appointment?								
No	113 (18.3)	37 (17.5)	71 (18.8)	78 (17.0)	31 (22.1)	59 (27.4)B	49 (12.9)	5 (23.8)
Yes	505 (81.7)	174 (82.5)	306 (81.2)	380 (83.0)	109 (77.9)	156 (72.6)	332 (87.1)A	16 (76.2)
General impression of home-based HIV care offered by ward-based outreach team members?								
Not a good initiative	29 (4.5)	14 (6.3)	14 (3.6)	16 (3.3)	11 (7.4)A	21 (9.3)B	8 (2.0)	01 (0.0)
Good initiative	617 (95.5)	210 (93.8)	378 (96.4)	462 (96.7)B	137 (92.6)	206 (90.7)	384 (98.0)A	251 (100.0)

Results are based on two-sided tests. For each significant pair, the key of the category with the smaller column proportion appears in the category with the larger column proportion.

Significance level (A, B, C): <0.05′′

Tests are adjusted for all pairwise comparisons within a row of each innermost sub-table using the Bonferroni correction.

More than half of the participants (*n* = 383; 57.5%) preferred monthly visits by CHWs to discuss HIV/AIDS-related issues. More participants who had been on treatment for <2 years and more participants who had never been told about CHWs and their role, indicated that they never wanted to be visited by CHWs to discuss HIV/AIDS-related issues, compared with their counterparts: 14.0% versus 7.0% (*p* < 0.01) and 18.2% versus 4.2% (*p* < 0.01), respectively. Overall, 16.3% of participants would not want CHWs to wear an official uniform during home visits.

This was especially true for participants who had not been informed about CHWs when they started treatment compared with those who had received CHW-specific information: 27.6% versus 9.6% (*p* < 0.01). Similarly, 28.8% of participants did not want CHWs doing home visits with branded cars. Interestingly, 81% of participants wanted to be traced by CHWs after missing a clinic appointment. This was also true for the majority (72.6%) of participants who had never been informed about CHWs' roles. Overall, 95.5% of participants thought that home-based HIV care is a good initiative that will help them.

### Home-based HIV services

On a scale of 0 to 10, where 0 is not at all and 10 is very much, participants were asked to rate different services that they thought CHWs should focus on. The most highly rated home-based HIV service offered by CHWs was screening participants for illnesses related to HIV, such as TB, followed by referral to a health facility when needed (Table 4). Adherence support was also a highly regarded service and participants rated CHWs' help in finding ways to remember to take medication every day third highest on the list (Table 4). HIV status disclosure was scored as the least important service. Whereas home delivery of medication was the third least important service, the SD was the highest, indicating the highest heterogeneity among responses.

**Table 4.** Home-Based HIV Services Offered by Community Health Workers

How much should CHWs focus on each of the following aspects of HIV care, when visiting you? On a scale of 0 to 10, where 0 is not at all and 10 is very much.	Number of responses	Mean	SD
Screen me for illnesses related to HIV such as TB	661	7.73	2.47
Refer me to a health facility when there is a need	579	7.67	2.59
Help me find ways to remember to take my medication every day	660	7.54	2.60
Educate me about side effects of HIV treatment	657	7.47	2.50
Teach me about my HIV medication	648	7.43	2.62
Help me with counseling after an HIV test	654	7.42	2.61
Educate and assist me with psychosocial well-being (cognitive, emotional, and spiritual strength combined with positive social relationships)	640	7.37	2.57
Deliver my medication at home	608	7.31	2.89
Educate me about the type of food I should eat to stay healthy	647	7.28	2.64
Assist me to be able to disclose my HIV status	656	7.25	2.66

TB, tuberculosis.

In univariate logistic regression analysis, no significant associations were evident regarding the rating of home-based HIV service offered by CHWs (Supplementary Table S7). The same was true for younger (<37 years) participants (71% vs. 75%, OR: 0.7, 95% CI: 0.4–1.0, *p* = 0.05; Supplementary Table S8). Participants who had been on treatment for ≥2 years rated home delivery of medication significantly higher than those who had been on treatment for <2 years (80% vs. 65%, OR: 2.4, 95% CI: 1.3–4.5, *p* < 0.01; Supplementary Table S9), whereas participants who had been informed about CHWs rated being referred to a health facility significantly higher than those who had not been informed (85% vs. 73%, OR: 2.1, 95% CI: 1.0–4.4, *p* = 0.05; Supplementary Table S10).

## Discussion

The aim of this study was to capture the voices of a representative group of PLWHIV regarding the acceptability of and their preferences for home-based HIV care offered by CHWs in Tshwane district. Emphasis was placed on participants' willingness to disclose their HIV status to different categories of health workers at both the household and clinic level, as well as perceptions about the various services offered.

## Disclosure

In clinical practice, great emphasis has been placed on disclosure of PLWHIV's status to family members, partners or friends, but little attention has been paid to disclosure to health care workers in general and CHWs in particular.^17,18^ The few studies that have assessed disclosure to health care workers have done so in the context of health facilities, with little or no focus on disclosure outside this environment.^18^ Results of this study demonstrate that participants were more comfortable disclosing their HIV status in a health facility compared with a community setting. In a study done on SA and Zambia, higher education levels were associated with lower judgmental beliefs.^19^ Interestingly, however, in our study willingness to disclose did not differ significantly between profession type (doctor, nurse, or CHW), but was rather determined by the environment (clinic vs. the community).

Approximately 20% of PLWHIV in this study were not willing to disclose their HIV status to health care workers in the community and can, consequently, not benefit from home-based HIV services. Participants who had been informed about HIV services offered by CHWs in the community were more willing to disclose their status to these health care workers, suggesting that a targeted information campaign could help to allay fears and improve engagement with this cadre of workers. This is especially important for PLWHIV who had been on treatment for a shorter duration, that is <2 years. Regional differences, independent of duration of treatment, also emerged and it is important to explore the reason for these differences through further qualitative research.

Attention should also be paid to recruitment strategies for CHWs. Although there are clear advantages of recruiting CHWs from a local area where they are knowledgeable about the local languages, logistics, socioeconomic realities, and life experiences of the community members they serve, this also poses problems.^20,21^ In this study, one of the main reasons participants were not willing to disclose their HIV status was distrust of neighbors.

Patient–health care provider trust has been defined as the expectation of the patient that the health care worker will act in their best interest. It is, however, unclear how patient–health care provider trust is modified in the context of pre-existing social relationships. Although it is worrying that a large proportion of participants were unwilling to disclose their HIV status to CHWs from their neighborhood, a large proportion would disclose their status to CHWs who come from other areas. There is, therefore, a need to revaluate the composition of WBPHCOTs and their geographic diversity to accommodate patients' needs.

### Home visit

In a study done in Tanzania, there were mixed feedback about helpfulness of community-based HIV services offered by CHWs.^22^ One of the most important findings of our study is that >95% of participants thought that home-based HIV care is a good initiative that will help them. This demonstrates that there is significant support for this intervention, but that specific issues need to be addressed to make the intervention acceptable to all. Although some PLWHIV view visits by CHWs as attracting unnecessary attention from community members,^9^ results of this study show that the vast majority (close to 90%) of participants want to be visited by CHWs. Importantly, however, ±28% of participants did not want CHWs to visit them with branded cars, whereas ±16% did not want CHWs to wear uniforms.

A systematic review on the frequency of clinic visit for PLWHIV showed that decreasing the number of clinics visits was effective in improving retention in care, but called for the introduction of community-based services to improve clinical outcomes.^23^ In this study, there was great variability in how often participants wished to receive home visits: from weekly, to monthly, to every 6 months. SA currently does not have enough CHWs to cover those in need of their services,^24^ so less frequent visits are the only realistic option.

Quarterly visits could still be effective since these visits should not be seen as a replacement of facility-based services, but rather as supplemental visits. Screening for opportunistic infections such as TB topped the list of services that participants preferred. PLWHIV, especially in the early stages of treatment, have an increased risk of opportunistic infections. In SA, TB remains the leading natural cause of death.^4^ In 2017, ±60% of patients with TB in SA were HIV positive and 56,000 of them died as a result of their infection.^25^ If PLWHIV are willing to be periodically screened by CHWs for TB, this could improve outcomes and reduce deaths.

The second ranked activity was being referred to a health facility when there is a need. Participants who had been informed about CHWs and their roles were more likely to value this service. This demonstrates the importance of the linkage between community-based services and facility-based services as well as provision of information about CHW roles to patients. Although women have been reported to be 33% more likely to have optimal adherence to ART compared with men in a South African study,^26^ men from our study appeared not to value assistance with identifying ways to remember their medication as high as women. Interestingly, participants who had been on treatment for longer and participants who were older rated delivery of medication higher than their counterparts. This information is valuable when considering ways in which to decongest clinics in a rational manner.

## Conclusions

In this study, home-based HIV care was largely accepted as an important and positive intervention. Participants did, however, have different preferences with regard to the practicalities of these visits. Differences were mostly determined by region, how long participants had been on ART, and whether they had been informed about the role of CHWs. Small differences were also evident between older and younger participants, as well as between genders. More work is needed to understand individual preferences and the extent to which they can be accommodated. Trust and disclosure of HIV status to CHWs remain central issues, which might require changes in recruitment and deployment of CHWs.

## Supplementary Material

Supplemental data

Supplemental data

Supplemental data

Supplemental data

Supplemental data

Supplemental data

Supplemental data

Supplemental data

Supplemental data

Supplemental data
