# Supplementary material for: Acceptability of Home-Based HIV Care Offered by Community Health Workers in Tshwane District, South Africa: A Survey
Source: AIDS Patient Care STDS. 2022 Feb 10;36(2):55–63. doi: 10.1089/apc.2021.0216 (PMC8861917; doi:10.1089/apc.2021.0216)
Supplement: Supplemental data [file Suppl_TableS1.docx]

Supplementary Table S1. Disclosure and home visits per sub district

|  | | **Region 1 (A)** | | **Region 2 (B)** | | **Region 3 and 4 (C)** | | **Region 5 and 6 (D)** | |
| --- | --- | --- | --- | --- | --- | --- | --- | --- | --- |
|  |  | N (%) |  | N (%) |  | N (%) |  | N (%) |  |
| Nurse in clinic | No | 2 (1.6%) | 1.6% | 6 (2.8%) | 2.8% | 23 | **11.1% A.B** | 6 | 7.8% |
|  | Yes | 122 | **98.4% C** | 206 | **97.2% C** | 185 | 88.9% | 71 | 92.2% |
| Nurse in community | No | 12 | 9.8% | 14 | 7.0% | 52 | **30.8%** | 31 | **44.3%** |
|  | Yes | 110 | **90.2%** | 185 | **93,0%** | 117 | **69,2%** | 39 | **55,7%** |
| Doctor in clinic | No | 3 | 2.4% | 4 | 1.9% | 7 | 5.3% | 2 | 2.9% |
|  | Yes | 120 | 97,6% | 209 | 98.1% | 126 | 94.7% | 68 | 97.1% |
| Doctor in community | No | 11 | 9.0% | 12 | 6.0% | 35 | 28.2% | 31 | 46.3% |
|  | Yes | 111 | 91.0% | 188 | 94.0% | 89 | 71.8% | 36 | 53.7% |
| CHW in clinic | No | 2 | 1.7% | 7 | 3.5% | 9 | 7.8% | 4 | 5.9% |
|  | Yes | 115 | 98.3% | 195 | 96.5% | 106 | 92.2% | 64 | 94.1% |
| CHW in community | No | 15 | 12.4% | 12 | 5.9% | 41 | 33.1% | 34 | 46.6% |
|  | Yes | 106 | 87.6% | 191 | 94.1% | 83 | 66.9% | 39 | 53.4% |
| CHW from your neighbourhood | No | 41 | 36.0% | 50 | 25.1% | 130 | 58.0% | 36 | 50.0% |
|  | Yes | 64 | 56.1% | 143 | 71.9% | 82 | 36.6% | 8 | 11.1% |
| CHW from area not close to your home | No | 3 | 2.4% | 47 | 21.2% | 41 | 18.1% | 19 | 22.9% |
|  | Yes | 121 | 96.8% | 156 | 70.3% | 171 | 75.7% | 37 | 44.6% |
|  | Declined to answer | 1 | 0.8% | 19 | 8.6% | 14 | 6.2% | 27 | 32.5% |
| Would you like to be visited by a CHW where you stay? |  | 0 | 0.0% | 2 | 0.9% | 1 | 0.4% | 3 | 3.6% |
|  | Yes. | 119 | 94.4% | 206 | 90.4% | 194 | 81.9% | 61 | 73.5% |
|  | No. | 7 | 5.6% | 16 | 7.0% | 37 | 15.6% | 9 | 10.8% |
|  | Declined to answer | 0 | 0.0% | 4 | 1.8% | 5 | 2.1% | 10 | 12.0% |
| How often should a CHW visit you? |  | 2 | 1.6% | 2 | 0.9% | 4 | 1.7% | 0 | 0.0% |
|  | Weekly | 11 | 8.7% | 27 | 11.8% | 99 | 41.8% | 7 | 8.4% |
|  | Monthly | 102 | 81.0% | 151 | 66.2% | 75 | 31.6% | 55 | 66.3% |
|  | 6-monthly | 6 | 4.8% | 28 | 12.3% | 25 | 10.5% | 6 | 7.2% |
|  | Yearly | 2 | 1.6% | 3 | 1.3% | 5 | 2.1% | 1 | 1.2% |
|  | Never | 3 | 2.4% | 17 | 7.5% | 29 | 12.2% | 14 | 16.9% |
| Should CHWs wear a uniform? | No | 18 | 14.5% | 25 | 11.1% | 51 | 22.1% | 7 | 8.5% |
|  | Yes | 103 | 83.1% | 188 | 83.2% | 162 | 70.1% | 66 | 80.5% |
|  | Declined to answer | 3 | 2.4% | 13 | 5.8% | 18 | 7,8% | 9 | 11.0% |
| Should CHWs come to your house with branded cars? | No response | 3 | 2.4% | 3 | 1.3% | 8 | 3.4% | 0 | 0.0% |
|  | No | 83 | 65.9% | 168 | 73.7% | 134 | 56.5% | 25 | 30.1% |
|  | Yes | 29 | 23.0% | 41 | 18.0% | 70 | 29.5% | 26 | 31.3% |
|  | Declined to answer | 11 | 8.7% | 16 | 7.0% | 25 | 10.5% | 32 | 38.6% |
| Should CHW to come to your house if you missed your clinic appointment? | No response | 0 | 0.0% | 1 | 0.4% | 6 | 2.5% | 2 | 2.4% |
|  | Declined to answer | 8 | 6.3% | 8 | 3.5% | 19 | 8.0% | 12 | 14.5% |
|  | No | 11 | 8.7% | 36 | 15.8% | 49 | 20.7% | 17 | 20.5% |
|  | Yes | 107 | 84..9% | 183 | 80.3% | 163 | 68.8% | 52 | 62.7% |
| General impression of the home-based HIV care offered by ward-based outreach team members? | No response |  |  |  |  |  |  |  |  |
|  | Good initiative | 125 | 99.2% | 211 | 92.5% | 215 | 90.7% | 66 | 79.5% |
|  | Not a good initiative | 0 | 0.0% | 11 | 4.8% | 11 | 4.6% | 7 | 8.4% |
|  | Declined to answer | 1 | 0.8% | 6 | 2.6% | 6 | 2.5% | 7 | 8.4% |
