# Supplementary material for: Acceptability of Home-Based HIV Care Offered by Community Health Workers in Tshwane District, South Africa: A Survey
Source: AIDS Patient Care STDS. 2022 Feb 10;36(2):55–63. doi: 10.1089/apc.2021.0216 (PMC8861917; doi:10.1089/apc.2021.0216)
Supplement: Supplemental data [file Suppl_TableS2.docx]

Supplementary Table S2. Significance Levels for Supplementary Table 1: Data Analysis per Sub District

| **Comparisons of column proportions^a^** | | | | | |
| --- | --- | --- | --- | --- | --- |
|  | | Region 1 (A) | Region 2 (B) | Region 3 and 4 (C) | Region 5 and 6 (D) |
| Nurse in clinic | No |  |  | A (0.010) B (0.005) |  |
|  | Yes | C (0.010) | C (0.005) |  |  |
| Nurse in community | No |  |  | A (0.000) B (0.000) | A (0.000) B (0.000) |
|  | Yes | C (0.000) D (0.000) | C (0.000) D (0.000) |  |  |
| Doctor in clinic | No |  |  |  |  |
|  | Yes |  |  |  |  |
| Doctor in community | No |  |  | A (0.001) B (0.000) | A (0.000) B (0.000) |
|  | Yes | C (0.001) D (0.000) | C (0.000) D (0.000) |  |  |
| CHW in clinic | No |  |  |  |  |
|  | Yes |  |  |  |  |
| CHW in community | No |  |  | A (0.001) B (0.000) | A (0.000) B (0.000) |
|  | Yes | C (0.001) D (0.000) | C (0.000) D (0.000) |  |  |
| CHW from your neighbourhood | No |  |  | A (0.001) B (0.000) | B (0.001) |
|  | Yes | C (0.004) D (0.000) | A (0.028) C (0.000) D (0.000) | D (0.000) |  |
|  | Declined to answer |  |  |  | A (0.000) B (0.000) C (0.000) |
| CHW not from neighbourhood | No |  | A (0.000) | A (0.000) | A (0.000) |
|  | Yes | B (0.000) C (0.000) D (0.000) | D (0.000) | D (0.000) |  |
|  | Declined to answer |  | A (0.017) |  | A (0.000) B (0.000) C (0.000) |
| Would you like to be visited by a CHW where you stay? | No response | .^a^ |  |  |  |
|  | a. Yes, they can come to my house | C (0.006) D (0.000) | C (0.050) D (0.001) |  |  |
|  | b. No, I don’t want them to come to my house |  |  | A (0.031) B (0.021) |  |
|  | c. Declined to answer | .^a^ |  |  | B (0.000) C (0.001) |
| How often should a CHW visit you at home? | No response |  |  |  | .^a^ |
|  | a. Weekly |  |  | A (0.000) B (0.000) D (0.000) |  |
|  | b. Monthly | B (0.020) C (0.000) | C (0.000) |  | C (0.000) |
|  | c. Once in 6 months |  |  |  |  |
|  | d. Yearly |  |  |  |  |
|  | e. Never |  |  | A (0.010) | A (0.001) |
| Should CHWs wear a  uniform? | No |  |  | B (0.009) D (0.040) |  |
|  | Yes | C (0.045) | C (0.006) |  |  |
|  | Declined to answer |  |  |  |  |
| Should CHWs come to your house with branded cars? |  |  |  |  | .^a^ |
|  | No | D (0.000) | C (0.001) D (0.000) | D (0.000) |  |
|  | Yes |  |  | B (0.021) |  |
|  | Declined to answer |  |  |  | A (0.000) B (0.000) C (0.000) |
| Should CHW to come to your house if you missed your clinic appointment? |  | .^a^ |  |  |  |
|  | Declined to answer |  |  |  | B (0.003) |
|  | No |  |  | A (0.021) |  |
|  | Yes | C (0.005) D (0.001) | C (0.027) D 0.008) |  |  |
| General impression of home-based HIV care offered by ward-based outreach team members? |  | .^a^ | .^a^ |  |  |
|  | a. Good initiative | B (0.038) C (0.009) D (0.000) | D (0.007) | D (0.044) |  |
|  | b. Not a good initiative | .^a^ |  |  |  |
|  | c. Declined to answer |  |  |  | A (0.029) |
| Results are based on two-sided tests. For each significant pair, the key of the category with the smaller column proportion appears in the category with the larger column proportion.  Significance level for upper case letters (A, B, C): 0.05 | | | | | |
| a. This category is not used in comparisons because its column proportion is equal to zero or one. | | | | | |
| b. Tests are adjusted for all pairwise comparisons within a row of each innermost subtable using the Bonferroni correction. | | | | | |
