# Supplementary material for: Acceptability of Home-Based HIV Care Offered by Community Health Workers in Tshwane District, South Africa: A Survey
Source: AIDS Patient Care STDS. 2022 Feb 10;36(2):55–63. doi: 10.1089/apc.2021.0216 (PMC8861917; doi:10.1089/apc.2021.0216)
Supplement: Supplemental data [file Suppl_TableS4.docx]

Supplementary Table S4. Significance Levels for Table 2 (HIV Status Disclosure to Nurses, Doctors and CHWs at a Facility and Community level)

|  | | Duration on Treatment | | 1.3 Gender | | Informed about CHWs and their role  n (%) | | |
| --- | --- | --- | --- | --- | --- | --- | --- | --- |
|  |  | <2 years (A) | ≥2 years (B) | Female (A) | Male (B) | No (A) | Yes (B) | Not sure (C) |
| Nurse in clinic | No |  |  |  | A (0.008) |  |  |  |
|  | Yes |  |  | B (0.008) |  |  |  |  |
| Nurse in community | No | B (0.000) |  |  |  | B (0.001) |  |  |
|  | Yes |  | A (0.000) |  |  |  | A (0.001) |  |
| Doctor in clinic | No |  |  |  |  |  |  | .^a^ |
|  | Yes |  |  |  |  |  |  | .^a^ |
| Doctor in community | No | B (0.000) |  |  |  | B (0.000) |  |  |
|  | Yes |  | A (0.000) |  |  |  | A (0.000) |  |
| CHW in clinic | No |  |  |  | A (0.035) |  |  |  |
|  | Yes |  |  | B (0.035) |  |  |  |  |
| CHW in community | No | B (0.000) |  |  |  | B (0.000) |  |  |
|  | Yes |  | A (0.000) |  |  |  | A (0.000) |  |
| CHW from your neighbourhood | No | B (0.035) |  |  |  | B (0.002) |  |  |
|  | Yes |  | A (0.005) |  |  |  | A (0.000) |  |
|  | Decline to answer |  |  |  |  | B (0.002) |  | B (0.001) |
| CHW not from neighbourhood | No |  |  |  |  |  |  |  |
|  | Yes |  |  | B (0.038) |  |  |  |  |
|  | Decline to answer | B (0.008) |  |  |  |  |  | B (0.009) |
| CHW = Community health worker  Results are based on two-sided tests. For each significant pair, the key of the category with the smaller column proportion appears in the category with the larger column proportion.  Significance level for upper case letters (A, B, C): .05 | | | | | | | | |
| a. This category is not used in comparisons because its column proportion is equal to zero or one. | | | | | | | | |
| b. Tests are adjusted for all pairwise comparisons within a row of each innermost subtable using the Bonferroni correction. | | | | | | | | |
