# Supplementary material for: Acceptability of Home-Based HIV Care Offered by Community Health Workers in Tshwane District, South Africa: A Survey
Source: AIDS Patient Care STDS. 2022 Feb 10;36(2):55–63. doi: 10.1089/apc.2021.0216 (PMC8861917; doi:10.1089/apc.2021.0216)
Supplement: Supplemental data [file Suppl_TableS5.docx]

Supplementary Table S5. Reasons for Not Disclosing HIV Status to Community Health Workers from the Same Neighbourhood

| Why wouldn't you disclose your HIV status to a CHW who is your neighbour? | n | % |
| --- | --- | --- |
| a. I don't trust my neighbours | 118 | 42.4 |
| i. I am afraid that they will tell others | 6 | 2.2 |
| ii. I do not like to talk about my personal things with my neighbours | 5 | 1.8 |
| iii. Other | 1 | 0.4 |
| b. I don't like to talk about my personal things with my neighbours | 87 | 31.3 |
| c. I am afraid that they will tell other people about my HIV status | 47 | 16.9 |
| d. Other | 14 | 5.1 |
