# Supplementary material for: Acceptability of Home-Based HIV Care Offered by Community Health Workers in Tshwane District, South Africa: A Survey
Source: AIDS Patient Care STDS. 2022 Feb 10;36(2):55–63. doi: 10.1089/apc.2021.0216 (PMC8861917; doi:10.1089/apc.2021.0216)
Supplement: Supplemental data [file Suppl_TableS6.docx]

Supplementary Table S6. Significance levels for Table 3 (Home Visit by a Community Health Worker)

|  | | Duration | | Gender | | Informed about CHWs and their role | | |
| --- | --- | --- | --- | --- | --- | --- | --- | --- |
|  |  | <2 years (A) | ≥2 years (B) | Female (A) | Male (B) | No (A) | Yes (B) | Don’t remember (C) |
| Would you like to be visited by a CHW where you stay? | No | B (0.029) |  |  |  | B (0.000) |  |  |
|  | Yes |  | A (0.029) |  |  |  | A (0.00) |  |
| How often should a CHW visit you at home? | Never | B (0.004) |  |  |  | B (0.000) |  |  |
|  | Weekly |  |  |  |  |  | A (0.00) |  |
|  | Monthly |  |  |  |  |  |  |  |
|  | 6-monthly |  |  |  |  |  |  |  |
|  | Once a year |  |  |  |  |  |  |  |
| Should CHWs wear a  uniform? | No |  |  |  |  | B (0.000) |  |  |
|  | Yes |  |  |  |  |  | A (0.00) |  |
| Should CHWs come  to your house with branded cars? | No |  |  |  |  |  | A (0.00) |  |
|  | Yes |  |  |  |  | B (0.003) |  |  |
| Should CHW to come to your house if you missed your clinic appointment? | No |  |  |  |  | B (0.000) |  |  |
|  | Yes |  |  |  |  |  | A (0.00) |  |
| General impression of home-based HIV care offered by ward-based outreach team members? | Not a good initiative |  |  |  | A (0.033) | B (0.000) |  |  |
|  | Good initiative |  |  | B (0.033) |  |  | A (0.00) |  |
| CHW = Community health worker  Results are based on two-sided tests. For each significant pair, the key of the category with the smaller column proportion appears in the category with the larger column proportion.  Significance level for upper case letters (A, B, C): .05 | | | | | | | | |
