# Supplementary material for: Acceptability of Home-Based HIV Care Offered by Community Health Workers in Tshwane District, South Africa: A Survey
Source: AIDS Patient Care STDS. 2022 Feb 10;36(2):55–63. doi: 10.1089/apc.2021.0216 (PMC8861917; doi:10.1089/apc.2021.0216)

Supplementary Table S10. Logistic Regression Analysis of Informed About Community Health Workers and Home-Based HIV Services Offered by Them


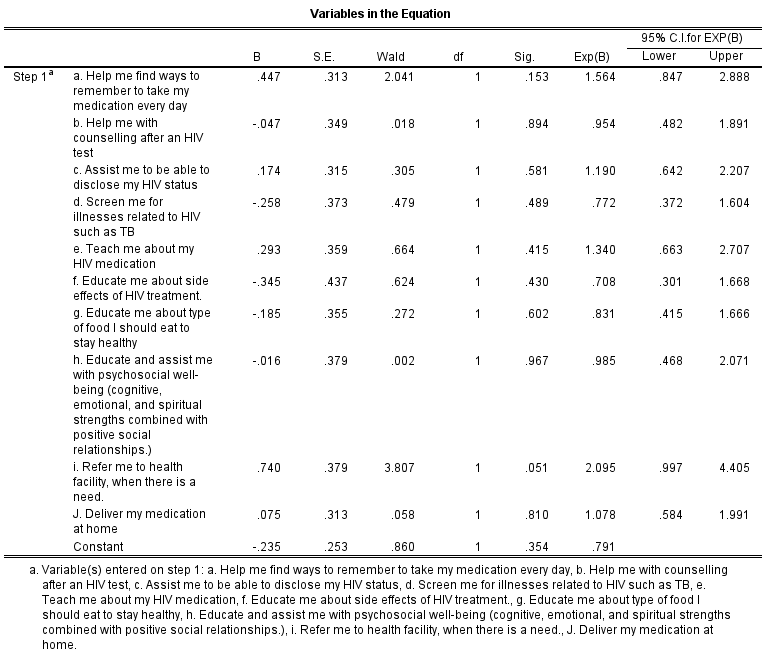

Supplement: Supplemental data [file Suppl_TableS10.docx]
